# Effects of Solvents, Emulsions, Cosolvents, and Complexions on Ex Vivo Mouse Myometrial Contractility

**DOI:** 10.1007/s43032-021-00576-5

**Published:** 2021-04-14

**Authors:** Christopher J. Hansen, Shajila Siricilla, Naoko Boatwright, Jackson H. Rogers, Melissa E. Kumi, Jennifer Herington

**Affiliations:** 1 Department of Pediatrics, Division of Neonatology, Vanderbilt University Medical Center, 2215B Garland Ave, 1125 Light Hall, Nashville, TN 37232 USA; 2 Department of Pharmacology, Vanderbilt University, Nashville, TN USA

**Keywords:** Emulsion, Isometric contractility, Uterus, Solvents, Tissue bath, Tocolytic

## Abstract

A great need exists to develop tocolytic and uterotonic drugs that combat poor, labor-related maternal and fetal outcomes. A widely utilized method to assess novel compounds for their tocolytic and uterotonic efficacy is the isometric organ bath contractility assay. Unfortunately, water-insoluble compounds can be difficult to test using the physiological, buffer-based, organ bath assay. Common methods for overcoming solubility issues include solvent variation, cosolvency, surfactant or complexion use, and emulsification. However, these options for drug delivery or formulation can impact tissue function. Therefore, the goal of this study was to evaluate the ability of common solvents, surfactants, cosolvents, and emulsions to adequately solubilize compounds in the organ bath assay without affecting mouse myometrial contractility. We found that acetone, acetonitrile, and ethanol had the least effect, while dimethylacetamide, ethyl acetate, and isopropanol displayed the greatest inhibition of myometrial contractility based on area under the contractile curve analyses. The minimum concentration of surfactants, cosolvents, and human serum albumin required to solubilize nifedipine, a current tocolytic drug, resulted in extensive bubbling in the organ bath assay, precluding their use. Finally, we report that an oil-in-water base emulsion containing no drug has no statistical effect beyond the control (water), while the drug emulsion yielded the same potency and efficacy as the freely solubilized drug.

**Supplementary Information:**

The online version contains supplementary material available at 10.1007/s43032-021-00576-5.

## Introduction

The myometrium serves as a therapeutic target for labor induction and management of dysmenorrhea, preterm labor, and postpartum hemorrhage. The discovery and development of novel compounds to regulate myometrial contractility routinely utilize an ex vivo organ bath assay [1, 2]. Unfortunately, the assay requires solubilization of the test compound into a water-based, ion-balanced, biological buffer, which is largely dependent on the compound’s polar character. An estimated 40% of approved drugs and nearly 90% of all drugs within the developmental pipeline consist of poorly soluble compounds [3].

Currently, there are several methods for overcoming poor compound solubility, including the modification of physicochemical properties of compounds through suitable salt forms, drug delivery systems (solvents, cosolvents, surfactants, emulsifiers, liposomes, and nanoparticles), and many others, as aptly reviewed by Kalepu and Nekkanti [3–5]. In the current study, we focused on the most common drug solubilization approaches (solvents, cosolvents, surfactants, complexions, and emulsions) to overcome poor water solubility in ex vivo organ bath contractility assays. The solubility of a compound is dependent on the polar relationship between it and the solvent [6]. Polar solvents, including ethanol (EtOH), isopropanol (IPA), methanol (MeOH), and water, dissolve ionic and polar compounds. Semi-polar solvents, such as acetone (ACE), acetonitrile (MeCN), dimethylacetamide (DMA), dimethylformamide (DMF), dimethyl sulfoxide (DMSO), and ethyl acetate (EtAc), can improve the miscibility of nonpolar compounds in polar solvents [4]. Typically, nonpolar solvents are not used in ex vivo or in vitro studies due to their high toxicity and poor water miscibility which commonly include hexanes and alkyl halides [7–10]. Surfactants contain both hydrophilic and hydrophobic groups that help solubilize water-insoluble compounds through the formation of micelles [11]. Emulsions are mixtures of two liquid phases, usually oil and water, in which one is dispersed into the other using an emulsifier, such as a surfactant, to increase its kinetic stability [11].

Currently, most reported studies using ex vivo organ baths to study myometrial contractility utilize water, DMSO, or EtOH to dissolve their compound(s) of interest [2, 12, 13]. To this end, we were not able to identify prior reports that examined the ability of other solvents and approaches to solubilize compounds in the ex vivo myometrial contractility organ bath assay without affecting myometrial contractility. Therefore, the goal of this study was to evaluate the effect of other solvents, cosolvents, detergents, complexions, and emulsions in ex vivo uterine contractility assays to provide the field with drug delivery options for overcoming issues in drug solubility.

## Materials and Methods

### Reagents

Solvents, surfactants, cosolvents, oils, and serum albumin were used from 100% stock unless otherwise noted. Acetone (A949), acetonitrile (A955), and ethyl acetate (E145) were purchased from Fisher Chemical. Isopropanol (I9516), dimethyl sulfoxide (D1435), ethanol (E7023), methanol (646377), N,N-dimethylformamide (D4551), human serum albumin (Al653), sesame oil (S3547), Cremophor EL (238470), nifedipine (N7634), Triton X-100 (T9284), and Tween 80 (P1754) were all purchased from Millipore Sigma. Polyethylene glycol 400 (B21992) and N,N-dimethylacetamide (22916) were purchased from Alfa Aesar. Lastly, vitamin E TPGS (HY-B0717) was purchased from MedChemExpress.

### Tissue Collection

Experiments involving mice received prior approval from Vanderbilt University Institutional Animal Care and Use Committee and conformed to the guidelines established by the National Research Council Guide for Care and Use of Laboratory Animals. Adult (8–12 weeks of age) CD-1® IGS mice (Charles River Laboratory) were housed in 12-h light:12-h dark cycles with free access to food and water. Copulation plugs following overnight breeding defined the first day of pregnancy, with normal delivery occurring on day 19.5. Mice were euthanized on gestation day 19 with an overdose of isoflurane followed by cervical dislocation. The uterus was excised via cesarean section. After removal of fetuses, placentas, and amniotic and endometrial membranes, the myometrium was used in ex vivo organ bath contractility assays.

### Surfactant, Cosolvent, and Complexation Preparation

Twofold serial dilutions (0.03125–0.5%) of the following were made in deionized (DI) water: Triton X, Tween 80, PEG 400, Cremophor EL, and vitamin E D-α-tocopherol polyethylene glycol succinate. A twofold serial dilution (0.266–4.25%) of human serum albumin was made in DI water to reflect its serum concentration [14]. A stock 0.1 M solution of nifedipine was made in 100% ethanol. Dilutions of the cosolvents, surfactants, and the complexion were measured out to 1 mL each in 1.5-mL glass vials and then heated to 37 °C via a warm water bath. Nifedipine was added such that the final concentration in each tested ratio was 0.1 mM. Solubility of each dilution was evaluated visually for the macroscopic appearance of precipitate. The concentration capable of fully or partially solubilizing nifedipine in solution was tested in an ex vivo organ bath assay.

### Emulsion Preparation

An oil in water (O/W) emulsion was prepared at a 10/70/20 ratio of oil, water, and vitamin E D-α-tocopherol polyethylene glycol succinate (TPGS). The non-ionic surfactant TPGS was chosen as an emulsifier based on its low critical micellar concentration (cmc = 0.02% w/w), lack of toxicity, and high hydrophilic-lipophilic balance value (HLB = 13.2) [15–17]. This was deemed suitable for immediate formation of O/W droplets and efficient dispersion of these droplets within the aqueous environment for stability throughout the experimental duration. The oil phase (sesame oil) was added to the water phase containing TPGS (1.8% w/v) and then emulsified using a homogenizer (VWR PowerMax AHS 200), at a fixed homogenization rate of 24,000 rpm for 2 min to reduce droplet size by shear stress. For emulsions containing the tocolytic drug nifedipine, the drug was first dissolved in acetonitrile and added continuously to the pre-emulsified O/W emulsion and homogenized for an additional 1 min under identical conditions. Acetonitrile was selected as the solvent based on both its low physiological effect on mouse myometrial tissue, as reported in this study, and efficacy in solubilizing nifedipine. The stability of each emulsion was evaluated visually for the macroscopic appearance of precipitate and separation of oil phase from water phase. Final concentrations of vitamin E TPGS and acetonitrile in tissue bath were 0.0013% w/v and 0.02% v/v, respectively.

### Ex Vivo Organ Bath Contractility Assay

The myometrium was cut into uniform longitudinal strips (1 cm × 0.5 cm × 0.1 cm) for use in organ bath isometric contractility assays as previously described [12, 13]. Briefly, myometrial strips were submerged into oxygenated (95% O_2_ and 5% CO_2_) and heated (37 °C) Radnoti organ baths containing pH 7.4 Krebs-bicarbonate solution (KBS) (136.7 mM NaCl, 4.7 mM KCl, 2.5 mM CaCl_2_, 25.2 mM NaHCO_3_, 1.5 mM MgCl, 1.8 mM NaH_2_PO_4_, and 15 mM glucose per liter of deionized water). Tissue was placed under 1*g* of tension and allowed to equilibrate for 1 h, during which rhythmic and spontaneous contractions developed. After recording spontaneous contractility, solvents were cumulatively added every 10 min in twofold serial dilutions for a final concentration of 0.025–1.000%. In separate experiments, the emulsion base (o/w emulsion without nifedipine), nifedipine emulsion, and nifedipine in solvent (MeCN) were added cumulatively every 10 min as 10-fold serial dilutions for a final concentration of 10 pM–0.1 mM

Contractions were recorded using PowerLab/8SP and analyzed using ADInstruments LabChart 7Pro software. Contractile activity was analyzed for amplitude (average cyclic height per 10 min), frequency (number of contractions per 10 min), and integral (AUC relative to both baseline and period of duration (~600 s)). All analyses were expressed as percent change from baseline spontaneous contractility.

### Statistical Analysis

The effect of solvents and emulsions on myometrial contractility utilized at least 3–5 mice (3–8 tissue strips per mouse) per group. Statistical analyses and graphic visualizations were performed with GraphPad Prism 6.0 software. Data are expressed as mean ± SEM. Dose-response curves were analyzed first for the preferred model: three-parameter versus four-parameter fit. The three-parameter fit was the preferred model for the solvent data, while a four-parameter log fit was preferred for emulsion data. Finally, the CRC was used to determine whether (1) one curve fits all the data sets using extra sum-of-squares *F* test and (2) the EC_50_ and/or *E*_max_ values differ between data sets. A two-way analysis of variance followed by a post hoc Fisher’s LSD test was used to determine significant differences between the % response for each concentration of a given solvent versus the control (KBS). A two-way analysis of variance followed by a post hoc Tukey test for multiple comparisons was used to determine significant (*p* ≤ 0.05) differences between the % response for each concentration of emulsion base, nifedipine emulsion, and nifedipine in solvent (MeCN).

## Results

### Exploration of Solvent Effects on Myometrial Contractility

**Fig. 1:**
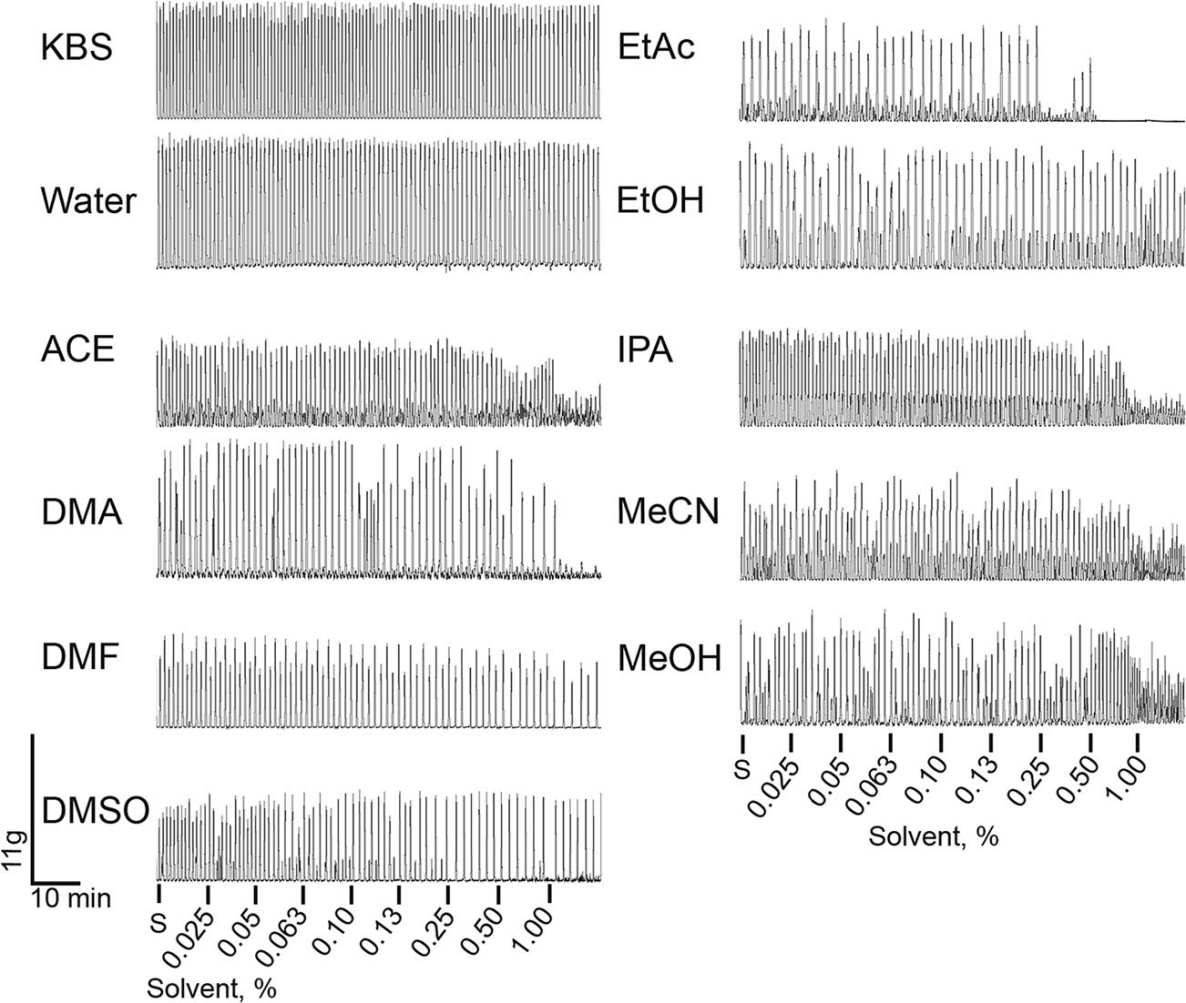
Representative contractility tracings of myometrial tissue treated with various solvents. Representative recordings of spontaneous contractility (S) prior to treatment with cumulative additions of solvents (0.025–1.0%)

**Fig. 2:**
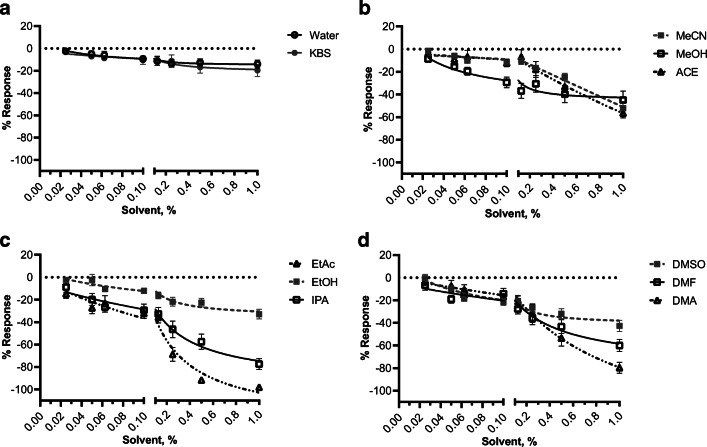
Effect of solvents on ex vivo myometrial contractile area under curve. Isometric tension recordings were analyzed for integration relative to baseline (AUC normalized to the treatment duration, ~600 s) for each cumulative addition of solvent. Concentration-response curves were visualized using a three-parameter nonlinear fit. Data is shown as mean ± SEM % response relative to baseline (spontaneous contractility, S) from 3 to 8 myometrial strips from d19 term pregnant mice (*N* ≥ 5). A two-way analysis of variance followed by a post hoc Fisher’s LSD test was used to determine significant differences between the % response for each concentration of a given solvent versus KBS and reported in supplemental Table 1

The effects of common solvents used to dissolve water-insoluble drugs were investigated on the contractility of myometrial strips from pregnant mice. Also investigated were controls, including the KBS used in the organ bath assay allowing examination of experimental duration on myometrial contractility, as well as water which is often used as the solvent for polar compounds. Representative recordings of spontaneous contractions (measured in grams of tension) prior to the cumulative additions of increasing concentrations of solvents and controls are shown in Fig. 1. After analyzing the area under the contractile curve (AUC; Fig. 2), which takes into account the contractile frequency, amplitude, and duration, we found the solvent with the least effect on myometrial contractility was EtOH, which was statistically insignificant (*p* > 0.05) from the control KBS at every concentration (Supplemental Table 1). Furthermore, ACE and MeCN had moderate effects on myometrial contractility, showing statistically significant differences compared to control only at 0.5% and 1% concentration, respectively. We observed that EtAc, IPA, and DMA had the greatest inhibition on myometrial contractility reaching ~30% inhibition at 0.1% (EtAc and IPA) and 0.25% (DMA), and maximum efficacy of greater than 80% inhibition.

**Fig. 3:**
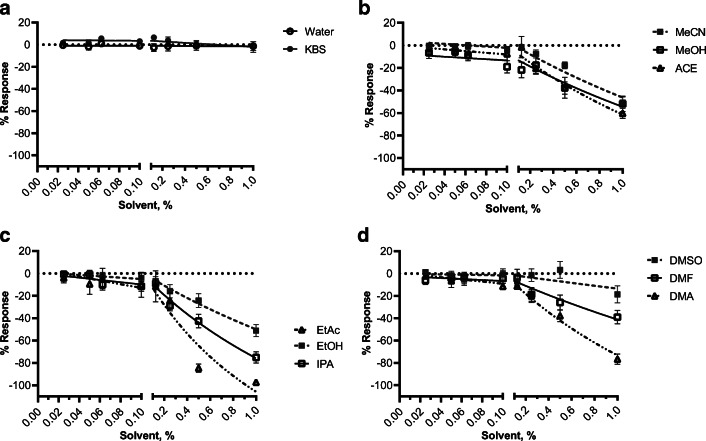
Effect of solvents on ex vivo myometrial contractile amplitude. Tissue contraction recordings were analyzed for average cyclic height (contractile amplitude) for each cumulative addition of solvent. Concentration-response curves were visualized using a three-parameter nonlinear fit. Data is shown as mean ± SEM % response relative to baseline (spontaneous contractility, S) from 3 to 8 myometrial strips from d19 term pregnant mice (*N* ≥ 5). A two-way analysis of variance followed by a post hoc Fisher’s LSD test was used to determine significant differences between the % response for each concentration of a given solvent versus KBS and reported in supplemental Table 1

**Fig. 4:**
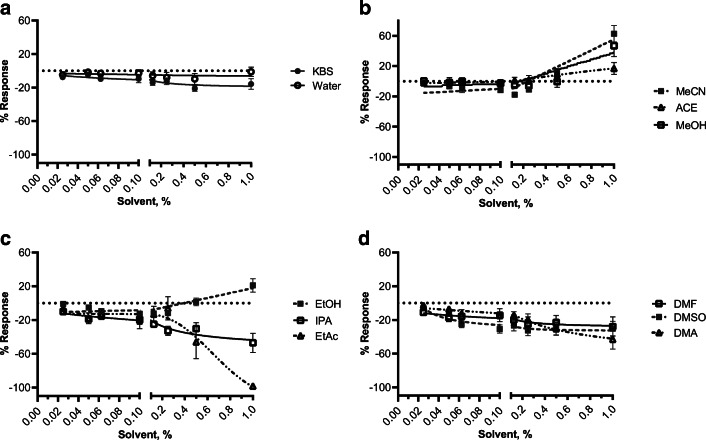
Effect of solvents on ex vivo myometrial contractile frequency. Tissue contraction recordings were analyzed for relative frequency per 10 min for each cumulative addition of solvent. Concentration-response curves were visualized using a three-parameter nonlinear fit. Data is shown as mean ± SEM % response relative to baseline (spontaneous contractility, S) from 3 to 8 myometrial strips from d19 term pregnant mice (*N* ≥ 5). A two-way analysis of variance followed by a post hoc Fisher’s LSD test was used to determine significant differences between the % response for each concentration of a given solvent versus KBS and reported in supplemental Table 1

We report that amplitude data (Fig. 3) is largely reflective of AUC data. Some of the least modulating solvents based on amplitude include EtOH and MeCN (~50% maximum inhibition) which is congruent to AUC. However, unlike the AUC, DMSO had the weakest effect on myometrial amplitude reaching only 19% maximum inhibition. The most modulating solvents, as a function of amplitude, include EtAc, DMA, and IPA (97%, 77%, and 75% inhibition, respectively) similar in their effect on AUC. We observed that while some solvents significantly (supplemental Table 1) decreased the contractile AUC at the highest concentrations of solvents examined in Fig. 2, there conversely appeared to be an increase in the contractile frequency at the same percentage. To this end, MeOH mildly inhibited myometrial contractility with regard to AUC but displayed ~50% stimulation of contraction frequency (Fig. 4). This trend is also observed for EtOH, MeCN, and ACE (21%, 63%, and 17%, respectively). Despite this finding, all other tested solvents (DMSO, DMA, DMF, EtAc, and IPA) show inhibition of contractile frequency. Like AUC and amplitude, EtAC caused the most inhibitory (98%) effect on contractile frequency.

The majority of tested solvents began inhibiting myometrial contractions between 0.125 and 0.25% concentrations (supplemental Table 1). With this considered, Table 1 shows the myometrial relaxant effect of solvents based on AUC at 0.1%, the concentration directly below 0.125%. At the timepoint (~2 h after the start of the experiment) that 0.1% of solvent was added in the organ bath assay, the control (KBS and water) treated myometrial tissues experienced 11% and 9% inhibition, respectively, in their contractile activity. Solvents with inhibitory contractile effects within the same range as these controls at the same experimental timepoint include the following: MeCN, EtOH, and ACE. However, solvents that had a significantly greater inhibitory effect at the 0.1% addition included the following: DMSO, EtAc, IPA, and MeOH (Table 1).

**Table 1:** Effect of solvents and controls on myometrial contractility at 0.1% concentration. The table lists the mean ± SD AUC relative to baseline at 0.1% concentration of the solvents referenced in Fig. 2

	% Response
Controls	
KBS	−10.55 ± 10.51
Water	−9.44 ± 10.14
Solvents	
MeCN	−11.92 ± 7.53
EtOH	−12.15 ± 5.18
ACE	−12.24 ± 10.98
DMF	−14.17 ± 15.94
DMA	−16.87 ± 8.90
DMSO	−21.40 ± 10.63
IPA	−29.40 ± 18.62
MeOH	−29.44 ± 15.51
EtAc	−32.21 ± 12.29
Emulsion	
MeCN Vit E-TPGS	−15.74 ± 13.94

Values shown are mean ± SD for contractile AUC at 0.1% solvent

### Determination of Surfactant, Cosolvent, and Complexation Effects in the Organ Bath

The most common pharmacological approaches to solubilize poorly water-soluble compounds include surfactant addition, cosolvency, and complexation [4, 18, 19]. We explored the effects of the common surfactants Tween 80, Triton X-100, vitamin E TPGS, and Cremophor EL at concentrations of 0.03125–0.5% v/v in an effort to fully or partially solubilize nifedipine, a water-insoluble compound that is clinically utilized as a tocolytic. Tween 80 was the only surfactant to fully solubilize the compound visually at the highest tested concentration (0.5% v/v), while the other surfactants studied only partially solubilized the drug. The commonly used cosolvent PEG 400 (0.5% v/v) and a water-soluble complexation of human serum albumin (4.25% w/v) were also tested in the organ bath at a maximum concentration required to fully or partially solubilize nifedipine visually. Polyethylene glycol 400 completely lacked the ability to solubilize nifedipine at even the highest concentration tested (0.5% v/v). Unfortunately, since the ex vivo organ bath contractility assay involves constant aeration with 95% O_2_/5% CO_2_, the surfactants and albumin in KBS cause extensive bubbling (Supplementary Figure 1) and thus lack the merit for further exploration.

### Investigation of Emulsion Effects on Myometrial Contractility

**Fig. 5:**
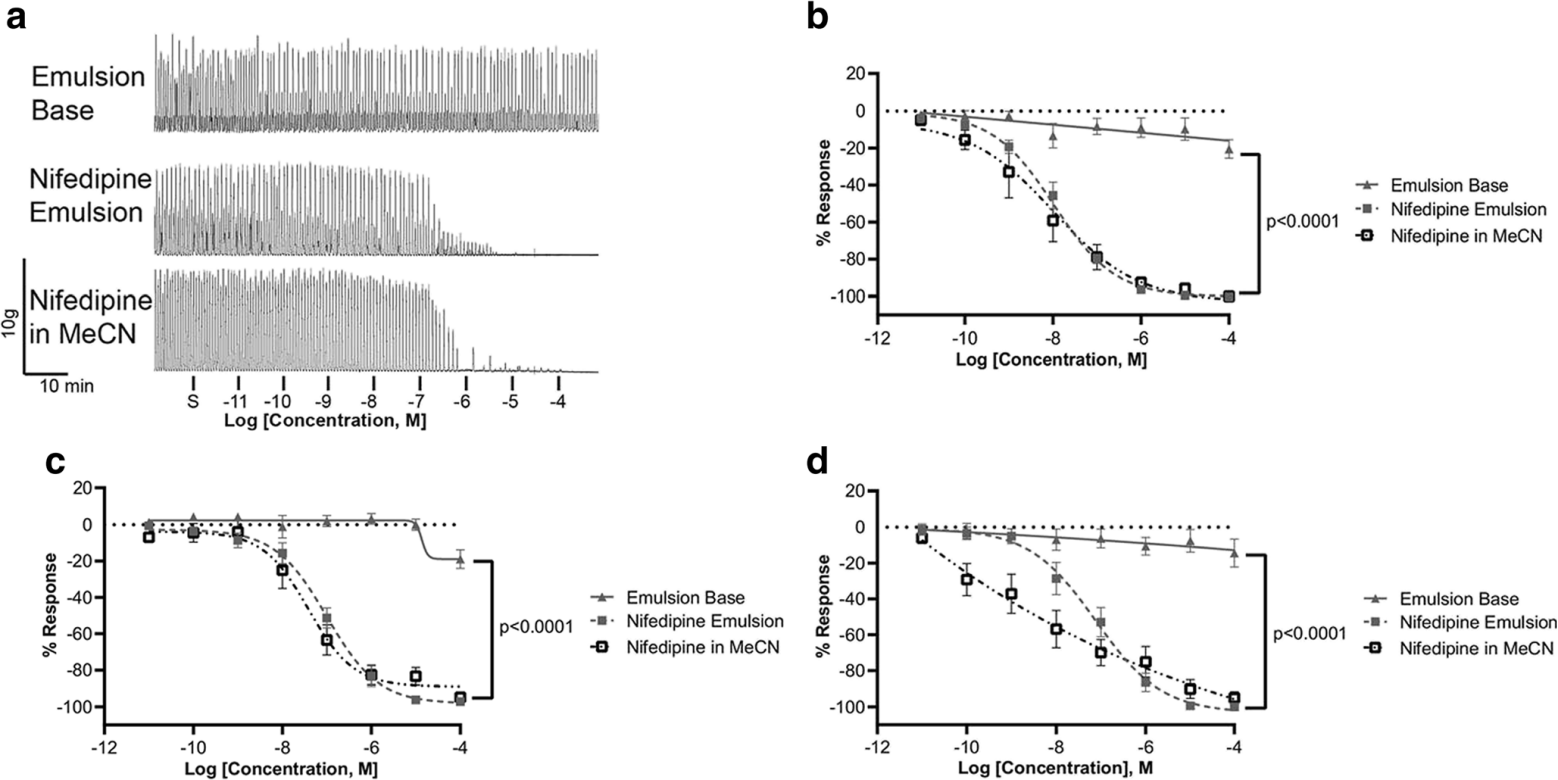
Utility of an emulsion as a formulation for ex vivo organ bath contractility assay. **a** Representative recordings of spontaneous contractility prior to cumulative treatment (10 pM–0.1 mM) every 10 min with either base emulsion, nifedipine (NIF) emulsion, or NIF in MeCN. Isometric tension recordings were analyzed for AUC (**b**), amplitude (**c**), and frequency (**d**). All concentration-response curves, including AUC, amplitude, and frequency, were visualized using a four-parameter nonlinear log fit. Data is shown as mean ± SEM % response relative to baseline (spontaneous contractility, S) from 3 to 4 myometrial strips from d19 term pregnant mice (*N* ≥ 3). Significant (*p* < 0.0001) difference between each fit line is indicated

An oil-in-water emulsion was prepared to examine whether this formulation strategy might be useful for ex vivo organ bath contractility assays. As a proof-of-principle, we also tested emulsions containing nifedipine and compared the drug’s efficacy and potency when administered as an emulsion versus freely dissolved in the solvent MeCN. Representative recordings of spontaneous contractions prior to the cumulative additions of increasing concentrations of nifedipine (emulsion and in MeCN) and control (emulsion base) are shown in Fig. 5a. We found that the myometrial tissue strips treated with increasing concentrations of the emulsion base (Fig. 5b–d) elicited a similar degree of inhibition at ~2 h after the start of the experiment as the controls KBS and water (Figs. 2, 3, and 4). After increasing concentrations of nifedipine were added to the organ bath, either as an emulsion or in a solvent, we observed similar inhibition (Fig. 5b–d) and no significant difference between the EC_50_ and *E*_max_ (Fig. 5b; *p* = 0.87). As seen in Fig. 5 c and d, the amplitude and frequency of emulsion data are congruent with that of AUC.

## Discussion

Early drug discovery, involving in vitro and ex vivo experiments, largely relies on aqueous-based assays. Ex vivo organ bath contractility assays using myometrial tissue remain an important screening tool for the discovery and development of effective, novel tocolytics and uterotonics [2, 12, 20–22]. It is crucial for the investigational compound to be in a homogenous solution within the organ bath buffer for accurate determination of a compound’s efficacy and potency. Currently, there are several solubilization methods; however, determining which technique to use depends largely upon its utility within a specific assay, taking into consideration tissue and cell effects, buffer composition, and test compound polarity [3, 23–27]. Many organ bath studies on a variety of muscle types have used various different solvents (DMSO, EtOH, MeOH, EtAc) to solubilize test compounds [28–32]. However, we were not able to identify prior reports that examined the ability of various solvents and solubilization approaches that adequately solubilize compounds in the ex vivo myometrial contractility organ bath assay without affecting myometrial contractility. Therefore, the present study provides insight into the effect of common solvents, surfactants, cosolvents, and emulsions on myometrial contractility in order to identify delivery methods for water-insoluble compounds with a minimal effect on myometrial contractility.

The present study indicates that EtAc, IPA, and MeOH dampen ex vivo mouse myometrial contractions at experimentally relevant concentrations, based on AUC and change in amplitude, compared to water and KBS controls. The most modulatory solvent reported in this study was EtAc, showing a nearly 100% inhibition of contractility at 0.5% v/v solvent. In contrast, the least modulatory solvent at the highest tested concentration was EtOH, having ~30% inhibition. Considering the inhibitory effect of the majority of studied solvents at concentrations greater than 0.125%, it appears that 0.1% serves as an appropriate threshold for solvent percentage within the myometrial contractility organ bath assay. The present study found that ACE, EtOH, and MeCN are the most desirable solvents for water-insoluble compounds. Conversely, DMSO, EtAc, IPA, and MeOH had an inhibitory effect statistically greater than the control solvents water and KBS and thus would not be desirable solvents for compounds in the myometrial organ bath assay. Amplitude data is congruent with AUC contractile data. However, we observed that MeOH, MeCN, EtOH, and ACE induce contractile frequency while inhibiting amplitude, which is supported by other studies [30].

Ethanol is a known uterine-relaxant. In fact, prior to its discontinued use due to detrimental fetal effects, ethanol was utilized clinically as a tocolytic agent [33–36]. However, the concentration required to achieve tocolytic benefit was 9.5–10% [35, 36]. Low-dose ethanol was found to be no more effective than placebo [37, 38] as a tocolytic to manage preterm labor. Similarly, the data presented here shows that EtOH causes no significant tocolytic effect in the organ bath assay for concentrations tested (≤1%), as analyzed by AUC.

Surfactants, cosolvents, and complexation agents are routinely added as solubilizing agents for in vitro assays [18, 19, 39]. For highly hydrophobic compounds, the concentration of solvent or surfactant required for solubilization can be detrimental to tissue activity or affect pharmacological assays [28, 39, 40]. Therefore, we tested the minimum concentration of surfactants, a cosolvent, and serum albumin required to solubilize nifedipine, a current tocolytic drug that is poorly soluble in water. As mentioned in the results, the bubbling experienced in the organ baths at the required concentrations discouraged further exploration. There have been reports of using perfluorodecalin to stabilize bubbles caused by the use of pulmonary surfactants, without affecting the assay system [41]. However, this approach to reduce bubbling in myometrial organ bath has not been reported, and the effect on myometrial contractility has yet to be established.

Emulsion-based delivery systems are frequently used as a formulation strategy for compounds with poor aqueous solubility in later stages of development [5, 27, 42–44]. In the present study, we investigated the potential of emulsion-based formulations as a delivery system for insoluble compounds in organ bath experiments. There are published reports on emulsions tested in organ baths on rat aortic tissue [45, 46]; however, this is the first report of emulsions being used as a solubilization method for poorly soluble compounds in an organ bath assay using mouse myometrial tissue. Importantly, we found that the base emulsion without drug was not different to that of water or KBS. Furthermore, it is seen that the emulsion of nifedipine in comparison to the freely solubilized nifedipine (in MeCN) is also not statistically significant, thus indicating that nifedipine in an emulsion is able to penetrate the tissue to elicit similar potency and efficacy. Here, we show that a base emulsion is aptly absorbed by tissue with little effect on normal function. This is an important note because solubility, though an obstacle, is not the goal in drug development. The ability of the tissue to absorb the drug, regardless of formulation, is more important. This suggests that emulsions may be effective delivery systems for poorly, or completely, water-insoluble compounds in this assay.

Isolated tissue bath contractility experiments are commonly used in pharmacological research on various tissues [2, 47–51]. While our study explores the effects of different solvents and emulsions on myometrial tissue, the known effect of each solvent may not be translatable to all tissue types explored in organ bath studies. Thus, there is a need to establish solvent effects for each tissue tested, similar to that of tissue selectivity for novel compounds, in organ baths [52–57]. Combinatorial solvent systems have also been used to maintain solubility for nonpolar compounds, such as 2:1 DMSO:EtOH [21]. Combination of solvents could alter myometrial tissue differently compared to individual solvents. Future studies that explore solvent combinations, and/or combinations with other additives mentioned here in tissue contractility assays, could benefit from the data presented in this study. Furthermore, the study described here was performed on mouse tissue, and therefore, the effects described here should be used as a starting point to assess solvents and drug solubility in studies on human myometrial strips, another tissue commonly used to assess the tocolytic or uterotonic efficacy and potency of compounds. Moreover, the emulsions tested here were done as a proof-of-concept to establish the possibility of usage; however, different compounds may require tailored emulsion formulations and longer stability studies depending on experimental parameters. Our emulsion was visually tested to be stable for 3–4 h, longer than the experiment duration using mouse myometrial tissue.

Our data provides a platform for the expanded understanding of the use of solvents and emulsion as drug delivery options in ex vivo myometrial organ bath systems. The work indicates that EtAc, IPA, and DMA have strong, contractile inhibitory effects, while ACE, EtOH, and MeCN have minimal effects on mouse myometrial tissue in organ bath assays at concentrations ≤ 0.1%. Furthermore, emulsions merit further exploration for their ability to maintain stable mixtures within this assay allowing compounds’ physiological effects to be explored.

## Supplementary Information

Fig. S1 **Representative images of surfactants, cosolvents, and complexion tested in an** ***ex vivo*** **organ bath assay.** Representative images of select surfactants, cosolvent, and complexation (human serum albumin) at the concentration (0.5% or 4.25%) required to partially or fully solubilize nifedipine macroscopically at 0.1mM. (EPS 9848 kb)

Table S1 **P-values for 2-way ANOVA comparing contractile AUC, amplitude, and frequency of solvents to control KBS.** A tabular view of the two-way analysis of variance followed and post hoc Fisher’s LSD test. Pink signifies a statistical (p-value <0.05) difference from that of control (KBS). (XLSX 14 kb)

## Data Availability

The data and material from this study are readily available from the senior author, Jennifer Herington.
